# A novel probe based on phenylboronic acid functionalized carbon nanotubes for ultrasensitive carbohydrate determination in biofluids and semi-solid biotissues†
†Electronic supplementary information (ESI) available: Fig. S1–S9, Table S1. See DOI: 10.1039/c5sc03992d


**DOI:** 10.1039/c5sc03992d

**Published:** 2015-11-19

**Authors:** Guosheng Chen, Junlang Qiu, Jianqiao Xu, Xu'an Fang, Yan Liu, Shuqin Liu, Songbo Wei, Ruifen Jiang, Tiangang Luan, Feng Zeng, Fang Zhu, Gangfeng Ouyang

**Affiliations:** a MOE Key Laboratory of Aquatic Product Safety/KLGHEI of Environment and Energy Chemistry , School of Chemistry and Chemical Engineering , Sun Yat-sen University , Guangzhou 510275 , P. R. China . Email: ceszhuf@mail.sysu.edu.cn ; Email: cesoygf@mail.sysu.edu.cn ; Fax: +86-020-84110845 ; Tel: +86-020-84110845

## Abstract

Carbohydrates are known to be involved in a wide range of biological and pathological processes. However, due to the presence of multiple hydroxyl groups, carbohydrate recognition is a particular challenge. Herein, we reported an ultrasensitive solid-phase microextraction (SPME) probe based on phenylboronic acid (PBA) functionalized carbon nanotubes (CNTs) for direct *in vitro* or *in vivo* recognition of carbohydrates in biofluids as well as semi-solid biotissues. The coating of the proposed probe possessed a 3D interconnected porous architecture formed by the stacking of CNTs. As a result, the binding capacity toward carbohydrates was excellent. The proposed approach was demonstrated to be much superior to most carbohydrate sensors, including higher sensitivity, wider linear range, and excellent qualitative ability in multi-carbohydrate systems. Thus, this approach opens up new avenues for the facile and efficient recognition of carbohydrates for important applications such as glycomics.

## Abstract 2

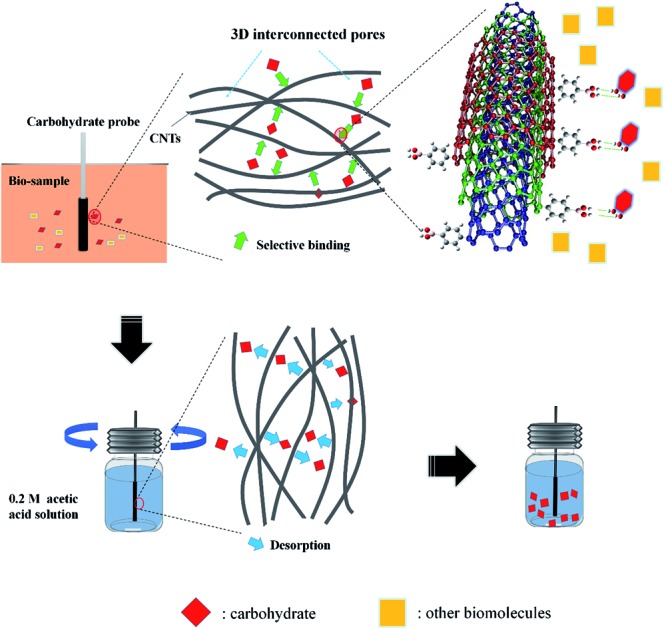
An ultrasensitive SPME probe based on phenylboronic acid functionalized CNTs is applied for direct *in vitro* or *in vivo* recognition of carbohydrates in biofluids as well as semi-solid biotissues.

## Introduction

Carbohydrates are known to be involved in a wide range of biological processes.^1^ Simultaneously, the concentration of carbohydrates in biological system is vital to several pathological processes. For example, diabetes mellitus, which is one of the biggest public health threats, demands continuous carbohydrate monitoring.^2^ Thus, precise determination of carbohydrates is necessary for not only fundamental researches but also clinical diagnoses.

Unlike nucleic acids, amino acids and lipids, determination of carbohydrates in aqueous solution is a tough challenge for chemists and biologists.^3^ Carbohydrates are hydrophilic species and therefore difficult to be extracted from water by traditional pre-treatment methods. As they contain multiple hydroxyl groups, they are also hydromimetic, blending easily into a background of water molecules.^4^,^5^ Synthetic receptors for specific carbohydrates recognition is a challenging yet highly impactful area of research.^6^–^9^ Phenylboronic acid (PBA) and its derivatives, known be able to rapidly and reversibly interact with 1,2- or 1,3-diols in aqueous media, are the most viable candidates for carbohydrate receptor design.^10^–^12^ However, the synthesis routes of receptors for carbohydrate sensors are usually complicated and tedious, and the efficiency and selectivity of the synthetic receptors, particularly ones that work in competitive solvents, remain a major challenge. The reasons for this are that the interactions of a receptor with the OH groups of a carbohydrate-derived substrate do not fundamentally differ from that with water molecules, which causes the cross-interference of the determination signals, and also the structural similarity of many carbohydrates, d-glucose and d-mannose, for example, differ in the configuration of only a single OH group on the ring.^5^ In addition, the determination principles mainly depend on the physicochemical signal changes of the receptor exposed to the sample, such as fluorescence,^13^–^15^ swelling/shrinking degree,^16^–^18^ diffraction^19^ and conductivity,^20^ and so on. It inevitably means that: (1) the limits of detection of carbohydrate sensors mostly range from hundreds of micromoles to millimoles per litre. Given this, the potential for application in unconventional body fluids containing low carbohydrate concentrations, such as interstitial fluid extracted by iontophoresis, tears, saliva and urine and at intracellular concentrations at the single-cell level in metabolomic studies,^21^–^24^ are infeasible. (2) The synthetic receptors are not capable of application for carbohydrate recognition in semi-solid or solid biological tissues, which are the main components comprising organisms.

**Scheme 1:**
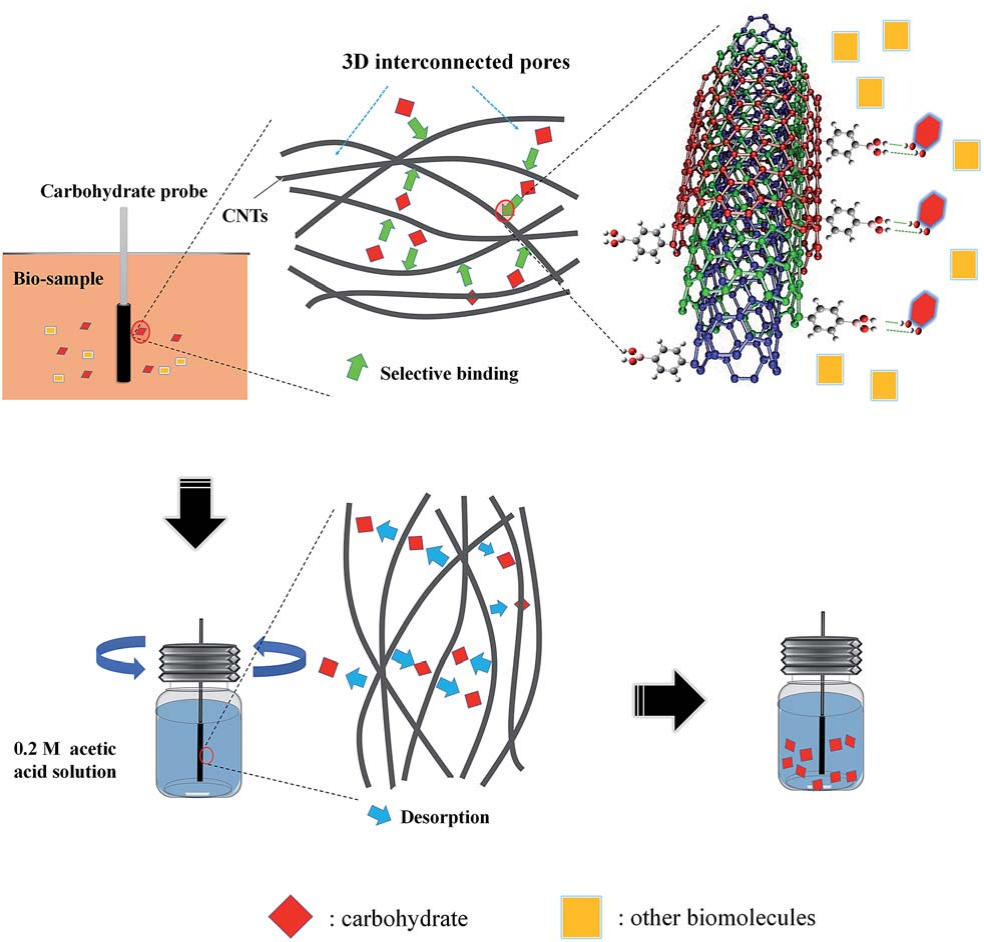
Representation of the carbohydrate recognition with the probe based on PBA functionalized-CNTs.

Carbon nanomaterials, such as carbon nanotubes (CNTs), have been explored extensively for carbohydrate-related biomolecule recognition in recent years.^25^,^26^ Pristine carbon nanomaterials are characterized by low solubility, thus the surface properties of CNTs must be tuned not just to improve their water solubility but also to enable these versatile nanomaterials to interact selectively with biological systems in aqueous systems. Chen *et al.* recently reported a review, which highlighted the strategies for synthesis of functionalized carbon nanomaterials and their applications in biosensing and biomedicine.^27^ As reported recently, functionalized CNTs could serve as excellent one-dimensional scaffolds for ligands, which can exhibit strong affinity towards lectin,^28^ glucose^9^ and glycan^29^ in aqueous systems. In addition, a novel nanocomposite consisting of 3-aminophenylboronic acid and CNTs was synthesized and an impedance-cell sensor was constructed.^30^ Herein, we fabricated an ultrasensitive SPME probe based on PBA functionalized CNTs, which enabled fast, quantitative and direct carbohydrate analysis in biofluids or semi-solid biotissues, by coupling with gas chromatography-mass spectrometry (GC-MS). Firstly, PBA functionalized-CNTs were synthesized and utilized as carbohydrate nano-receptors. The hybrid, containing an appropriate ratio of nano-receptors to other biocompatible and acid resisting polyacrylonitrile (PAN) groups, was attached to a pretreated quartz fiber through dip-coating method, forming a novel pH-controlled capture/release miniature probe for selective capture of carbohydrates. Owing to the 3D interconnected architecture formed by the stacking of PBA functionalized-CNTs in the coating, the proposed probe possessed excellent binding capacity toward carbohydrates (the enrichment factors were as high as 151). Simply by adjusting the pH of the eluent, this proposed probe was feasible to couple with GC-MS for carbohydrate separation and detection, which dexterously avoided the cross-interaction effect and simultaneously greatly improved the sensitivity for carbohydrate recognition (Scheme 1). Interestingly, the proposed probe was suitable to identify and differentiate carbohydrates in a multi-carbohydrate system. Moreover, this carbohydrate probe was successfully applied to determinate glucose in bovine serum and human urine without any expensive enzymes or tedious pretreatment procedure. Importantly, the excellent biocompatibility and mechanical strength of the probe made it possible to directly immerse the probe into semi-solid biological tissues (plant leaf and stem) for *in vivo* carbohydrate recognition and continuous carbohydrate monitoring.

## Results and discussion

### Synthesis and characterization of the receptor

**Fig. 1:**
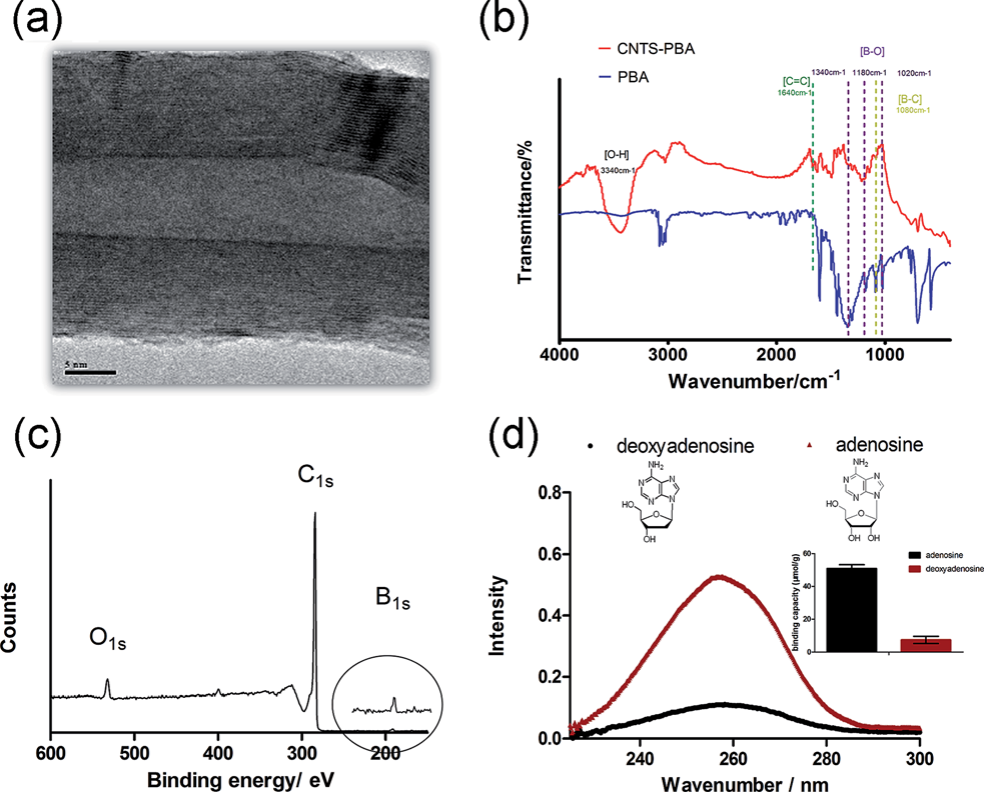
High TEM image (A), IR spectra, (B) and entire XPS spectrum (C) of the synthesized PBA functionalized-CNTs; (D) the selectivity of PBA functionalized-CNTs toward *cis*-diol.

Given the ultrahigh ratio of surface area to volume of carbon nanotubes (CNTs), and the extreme sensitivity of their surface atoms to any surface reaction events, functionalization of CNTs was used as the starting point. Herein, PBA functionalized-CNTs, with an external diameter about 40 nm (Fig. 1A and S1†), were synthesized and used as carbohydrate receptors. IR spectra (Fig. 1B) and XPS (Fig. 1C and S2†) confirmed the presence of PBA groups on CNTs. The affinity of the PBA functionalized-CNTs towards diol units was first confirmed using adenosine as a test compound, which contains a pair of *cis*-diol groups and it has UV absorbance at about 260 nm. Deoxyadenosine was used as an interferent, which also has UV absorbance at about 260 nm while containing no *cis*-diol moiety. As shown in Fig. 1D, the PBA functionalized CNTs exhibited excellent selectivity toward adenosine, and the binding amount was depended on the exposure time (Fig. S3†). In addition, the binding capacity toward adenosine was measured to be 50.9 ± 2.3 μmol g^–1^. These results demonstrate that the synthesized receptor showed excellent selectivity toward *cis*-diol.

### Binding capacity toward diol of the probe

**Fig. 2:**
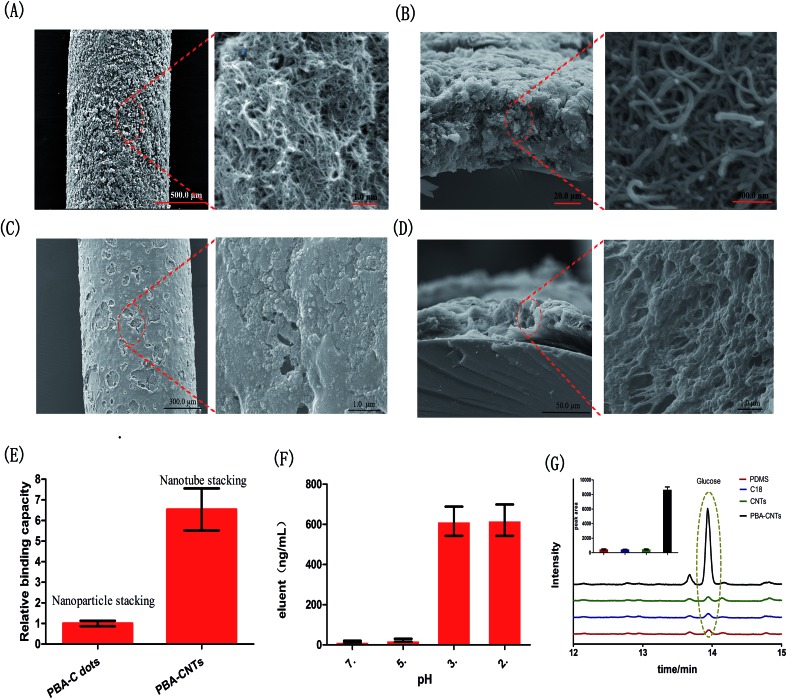
TEM images of the surface (A) and cross section (B) of the PBA functionalized-CNTs probe; TEM images of the surface (C) and cross section (D) of the PBA functionalized-carbon dots probe. It could be observed that no 3D interconnected pores were formed in the PBA functionalized-carbon dots probe; (E) the proposed probe with nanotube stacking possessed higher binding capacities than that based on PBA functionalized-carbon dots (nanoparticle stack). Note: the two kinds of probes were prepared in the same way; (F) concentrations of glucose in eluent at different pH (exposure time: 40 min); (G) comparison of the binding capacities of the PBA functionalized-CNTs probe with other widely used probes and the non-PBA functionalized probe.

There are two probe design challenges: (1) to apply an appropriate auxiliary for fixing the receptor on the solid substrate (quartz fiber); (2) to prevent the auxiliary material from covering the receptor, otherwise, the covering auxiliary could block binding sites of the receptor. Herein, PAN (*M*_w_ = 150 000, dissolved in dimethylformamide 1 : 10, v/v), which is regarded as a biocompatible and acid resisting polymer,^31^–^33^ was selected as the auxiliary for attaching the receptor to the pretreated quartz fiber through a dip-coating method, while dimethylformamide was utilized as it evaporates at high temperature (120 °C, 40 min) and so is suitable to create a porous structure of the coating (Fig. 2A). In addition, as seen in Fig. 2A and B and S4,† the stacking of CNTs could form 3D interconnected pores compared with the nanoparticle stacking (we fabricated another PBA functionalized-carbon dots based probe as a nanoparticle stacking probe model, which was shown in Fig. 2C and D). This architecture possessed high specific surface area and facilitated mass transfer in the coating, which greatly enhanced the availability of PBA groups (Fig. 2E). Meanwhile, owing to the reversible binding of PBA groups-diol unit^10^ and the high acid resisting ability of PAN, the bound diol unit was able to be released for further qualitative or quantitative analysis simply by adjusting the pH of the eluent (Fig. 2F). As shown in Fig. 2G, the extraction efficiency of the proposed probe toward diols was much superior to that of other probes widely used in biological analysis, including polydimethylsiloxane (PDMS) and C18.^34^–^37^ In addition, the comparison result between the PBA functionalized probe and non-PBA functionalized probe indicated that the extraction performance of the proposed probe was substantially due to the PBA groups in the coating, which demonstrated that the PBA groups in the probe were available and provided a specific scaffold for carbohydrate binding.

### Specificity of the probe toward carbohydrates

**Fig. 3:**
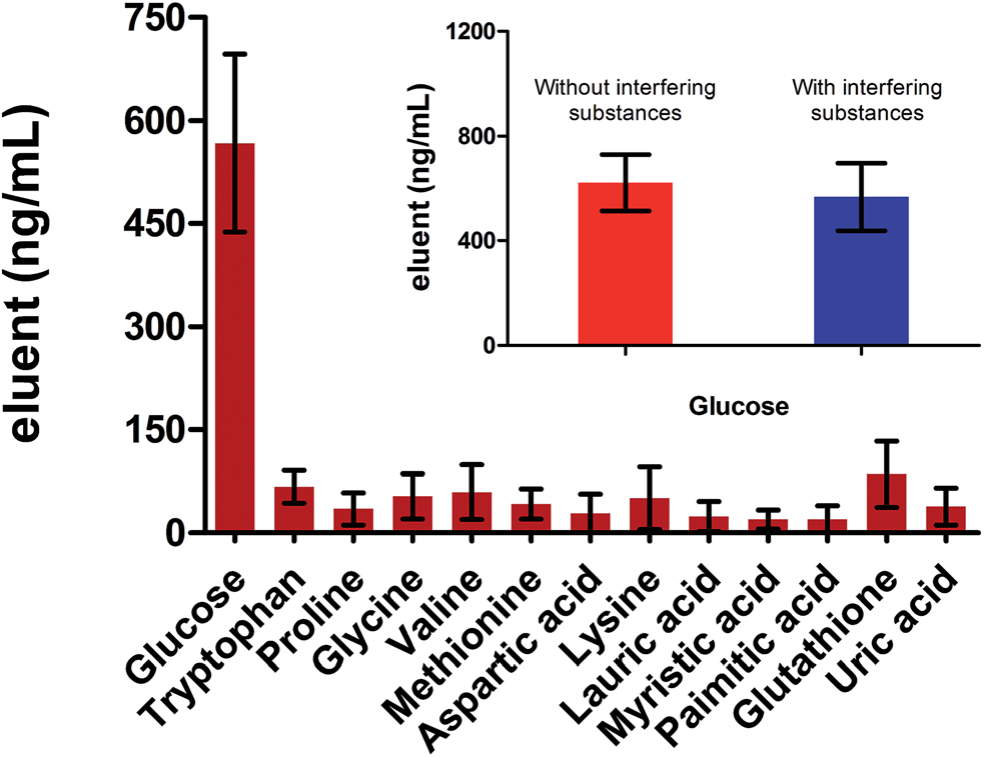
The response of the probe toward various potential interfering substances. The mixture, which contained glucose, various amino acids, aliphatic acids, glutathione and uric acid (concentrations = 10 μM), was extracted by the probe for 40 min. The result showed that the probe presented specific selectivity towards carbohydrate (glucose). The inset shows the response of the probe toward glucose in the solution without and with interfering substances.

Selectivity is a critical parameter to evaluate the performance of the probe. We firstly studied the response of the proposed probe toward potential interfering substances coexisting in biofluids, including various amino acids, aliphatic acids, glutathione and uric acid. As shown in Fig. 3, the extraction capacity of the probe toward these substances was negligible. The exclusive extraction of carbohydrate (glucose) resulted from the following reasons. Firstly, the carbohydrate possessed specific multiple hydroxyl groups structure while these coexisting substances have no such special structure. Secondly, as demonstrated in Fig. 1D and 2, the probe provided a specific scaffold for the diol unit, especially for the *cis*-diol. Thus, carbohydrates, which possess various 1,3 or 1,2 *cis*-diol units, could be easily captured by the scaffolds.

### Glucose recognition in PBS

**Fig. 4:**
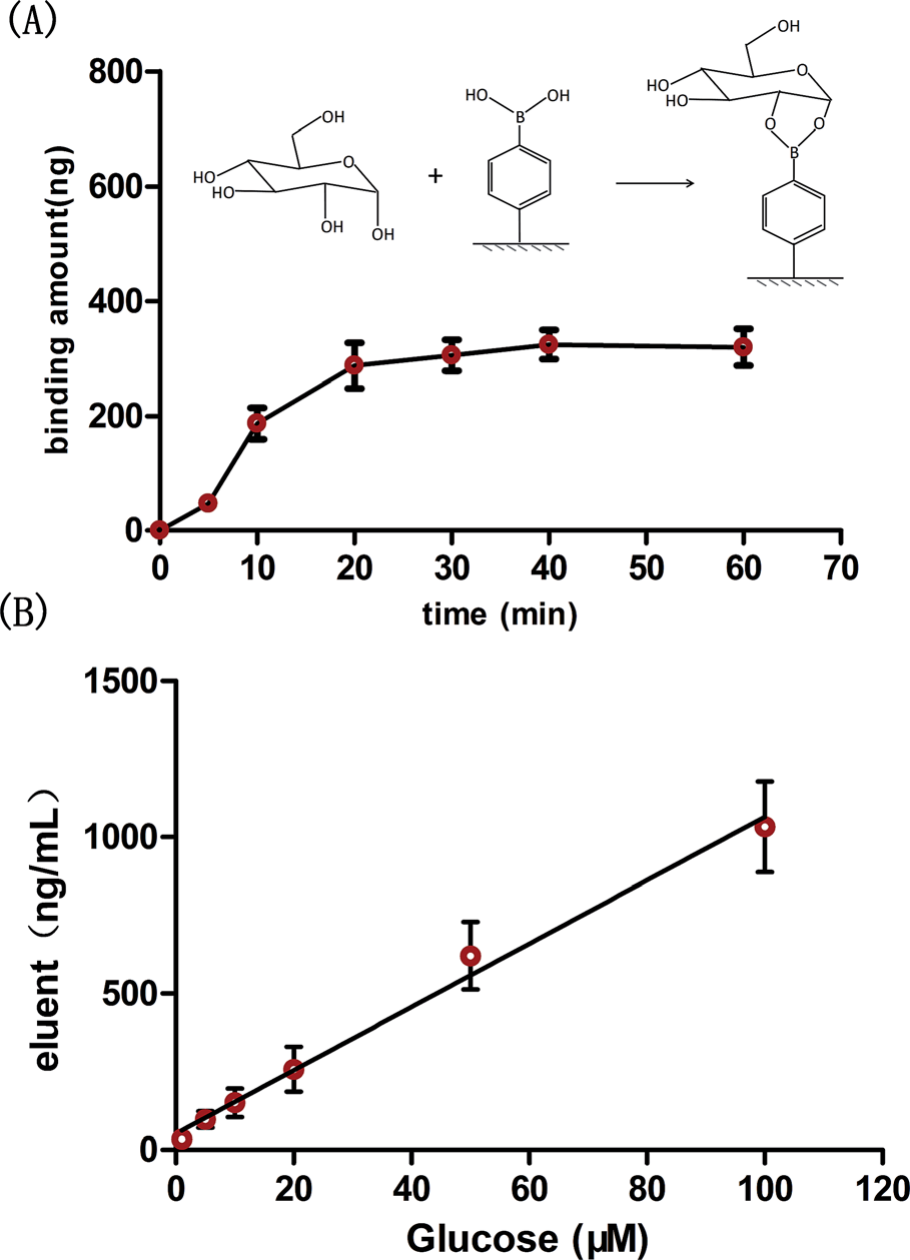
(A) Binding kinetics of the proposed probe toward glucose in PBS solution (100 μM glucose); (B) the wide linear range of glucose in PBS solution.

The performance of the prepared probe for carbohydrate assay was firstly evaluated in phosphate buffer solution (PBS). Glucose, a typical monosaccharide, was used as the carbohydrate model and 0.2 M acetic acid (pH ≈ 2) was selected as the eluent. As shown in Fig. 4A, the binding reaction was completed within 20 min. Such a good performance was due to the 3D interconnected pores and fixed orientation of the PBA molecules within the probe, which facilitated efficient mass transfer and complexing simultaneously.

Under the optimized conditions, the probe was applied for glucose determination in PBS solution. Due to the enrichment effect of the probe (see below) and the high resolution detector, the linear range of the probe was found to range from 1 to 100 μM for glucose determination (Fig. 4B), with a limit of detection of 0.12 μM (signal-to-noise ratio of 3). To our knowledge, the linear range and the detection limit of the proposed probe were much better than of most previous boronic acid based sensors (Table S1†). A higher sensitivity for glucose assay is important not only in low concentration biofluids, such as tears, saliva and urine, but also in high glucose level biofluids, such as blood (several to tens of millimoles per liter). The reason is that highly sensitive probes would allow a sufficient sample dilution during assay, which can effectively reduce interference from the complicated matrix (blood/serum).^38^ It is noteworthy that the whole assay procedure, consisting of binding, elution and detection, required less than 2 h. In addition, the reusability of the probe was evaluated. It was shown that the extraction efficiency did not significantly change even after 20 times reuse (Fig. S5†).

### Multi-carbohydrate recognition in PBS

**Fig. 5:**
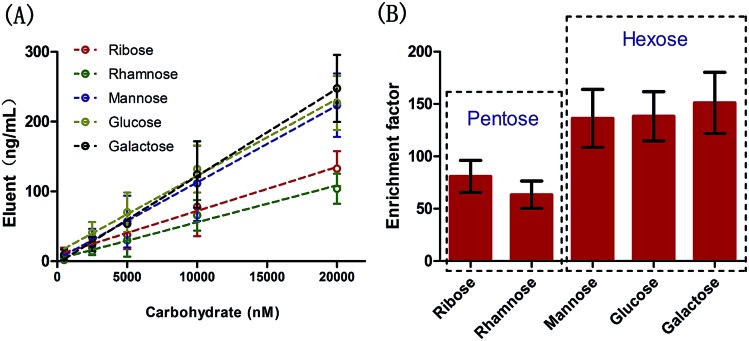
The linear ranges (A) and enrichment factors (B) of multi-carbohydrates in PBS solution using the proposed probe.

Carbohydrates are one of the most abundant molecules that comprise human life and many kinds of carbohydrate are present. The approaches based on sensors for carbohydrate recognition have some drawbacks, one is that it is not capable of qualitative or quantitative recognition of multi-carbohydrate in biofluids. In parallel with the emergence of glycomics, novel assays for fast and accurate carbohydrate recognition in multi-carbohydrate biofluids is required.^39^–^42^ Here, owing to excellent separation ability of chromatography as well as the high resolution of mass spectrometry, the proposed probe coupled with GC-MS was proved to be feasible to recognize different carbohydrates in a multi-carbohydrate PBS solution, with linear ranges from 0.5 to 20 μM (Fig. 5A), and much superior to the low qualitative or quantitative ability of the previous boronic acid based sensors.

Owing to the unique 3D interconnected architecture in the coating, the enrichment factors, defined as the ratio of the carbohydrate concentrations in probe and in matrix, were measured to range from 63 to 151 (Fig. 5B). Generally, the probe showed higher enrichment capacity toward hexoses than pentoses. The reasons we propose are: (1) PBA can bind with *cis*-1,2- or 1,3-diols to form a diol–phenylboronate complex with either five- or six-membered ring systems, respectively,^43^,^44^ (2) the binding amount is largely dependent on the probability of directional collision. From this point, hexoses, which have an additional hydroxy unit compared with pentoses, should more readily complex with PBA. It was noteworthy that no selectivity against structural similar carbohydrates, such as mannose, glucose and galactose, was required in this approach since the probe was easy to couple with GC-MS.

### Assay in serum and urine

Given the simplicity and ultra-sensitivity of carbohydrate determination with the proposed probe, the approach can be viewed as being ideal for monitoring carbohydrates in real biofluids. As a proof of concept, bovine serum was firstly used as a biofluid model. Considering the high glucose level in serum, 15 μL bovine serum was diluted 100 times with the PBS solution, and then was transferred into a 2 mL vial. The probe was directly immersed into the serum for glucose determination without any expensive enzymes or tedious pretreatment procedure. As shown in Table 1, the glucose level obtained by the proposed probe was in good agreement with the values measured by a commercial blood glucose monitor. Benefiting from the ultrasensitive property of the proposed approach, the probe was also successfully applied for glucose determination in urine, typically a trace glucose containing sample (Table 1).

**Table 1:** Glucose assay in bovine serum and human urine

Sample^*a*^	S-1	S-2	S-3	Average
Serum	4.6 mM	5.7 mM	6.9 mM	5.7 mM
Urine	16.0 μM	16.2 μM	17.7 μM	16.6 μM

^*a*^The average detected concentration of glucose in serum was 5.7 mM with the proposed probe, which was in good agreement with the values measured by a commercial blood glucose monitor (5.9 mM). Notably, glucose at a very low level (16.6 μM) was detectable using the proposed probe.

### *In vivo* carbohydrate recognition

**Fig. 6:**
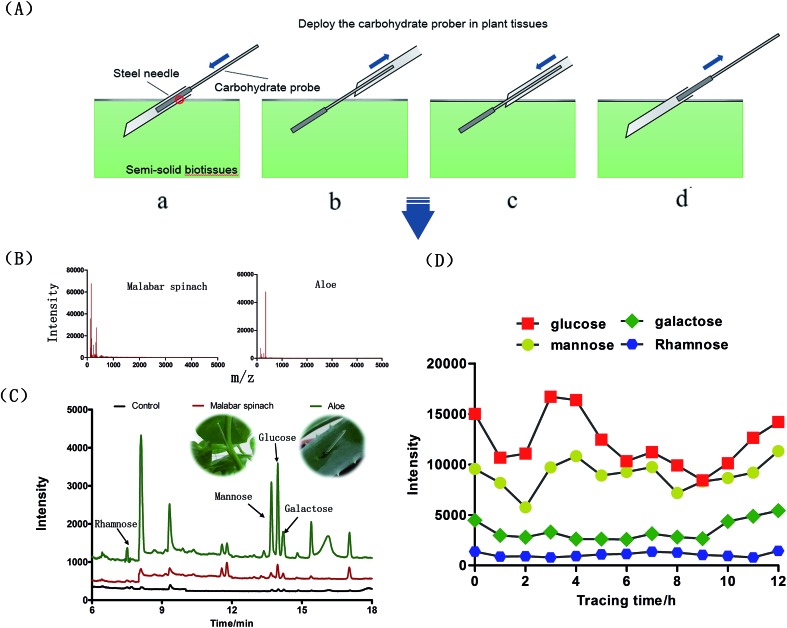
(A) The *in vivo* sampling procedure in plant tissues. The carbohydrate probe is deployed under the guidance of a steel needle (a), removal of the steel needle and exposure of the probe to the carbohydrate in plant tissues (b), the steel needle is carefully put back in the plant tissue at the end of sampling (C), the carbohydrate probe is removed (d). The probe was readily inserted into or removed from plant tissues; (B) biological macromolecules analysis in the eluent with MALDI-TOF MS; (C) carbohydrate assay in the stem of Malabar spinach and leaf of aloe. The signals of the corresponding carbohydrate were apparent; (D) *in vivo* continuous carbohydrate monitoring in aloe leaf using the proposed probe.

Previous fully-implantable sensors, embedded in the body for carbohydrate recognition, are seriously challenged mainly due to the insufficient physicochemical signal intensity required for transdermal detection.^45^ Fortunately, the proposed probe could overcome this tough challenge due to the different detection principle. Thus, besides the above *in vitro* analysis, the other major novel application of the proposed probe was *in vivo* carbohydrate recognition in semi-solid biotissues, such as plant stem and leaf. Fig. 6A briefly presents the *in vivo* sampling procedure in plant tissues. To achieve this goal, the probe should be capable of resisting the adhesion of biological macromolecules on the surfaces. Otherwise, the adhered biological macromolecules would block the binding sites of the probe and alter the ionization efficiencies when the macromolecules were desorbed in the desorption solvents. It was demonstrated through MALDI-TOF MS that no macromolecules were present in the eluent of probe exposed in aloe leaf or Malabar spinach stem for 30 min (Fig. 6B and S6†). Moreover, the excellent mechanical strength of the probe, which could be observed in ESI movie 1,† make it suitable for direct immersion into semi-solid biotissues. Regarding the carbohydrates detected in this study, glucose was the main monosaccharide detected in Malabar spinach while rhamnose, mannose, glucose and galactose were detected in aloe without any plant sacrifice (Fig. 6C). The abundant monosaccharide species detected in aloe leaf with the proposed assay was in good agreement with the previous analysis after several tedious pretreatments,^46^,^47^ which demonstrated the feasibility for *in vivo* analysis. Finally, the probe was used for noninvasive and long-term *in vivo* continuous carbohydrate monitoring in aloe leaf (Fig. 6D).

## Conclusions

In summary, a novel SPME probe based on PBA functionalized-CNTs is proposed for fast and ultrasensitive determination of carbohydrates in biofluids and semi-solid biotissues. The proposed approach was demonstrated to be much superior to previous carbohydrate sensors. It exhibited several significant advantages, including higher sensitivity, wider linear range, and excellent qualitative ability. Unlike the method based on carbohydrate sensors, no complicated and tedious synthesis route of the receptor was needed in the proposed approach. Moreover, the probe was capable of direct *in vitro* or *in vivo* determination of multi-carbohydrates in complex real sample matrices, and the total analysis procedure was time-efficient (less than 2 h). Notably, the preparation approach of the probe can be applicable to other nanomaterials. In conclusion, this approach opens up new avenues for facile and efficient tracking and recognition of carbohydrates in bio-samples and could be a promising approach for important applications such as glycomics.

## Experimental

### Reagents and materials

Multi-walled carbon nanotube (MWCNTs), ethylenediamine, *N*,*N*′-diisopropylcarbodiimide (DIC), *N*,*N*-diisopropylethylamine (DIEA), *N*-hydroxysuccinimide (NHS), dichloromethane (DCM), dimethylformamide (DMF), anhydrous tetrahydrofuran (THF) and all analytes for saccharides detection experiments were purchased from Aladdin Reagent (Shanghai, China); thionyl chloride (SOCl_2_) was obtained from Thermo Fisher Scientific (Guangzhou, China); 4-carboxyphenylboronic acid (4-CPBA), polyacrylonitrile (PAN), sodium borohydride and methylimidazole were purchased from J&K Scientific (Beijing, China); nitric acid, ethanol, diethyl ether, acetic acid and acetic anhydride were from Guangzhou Reagent Company (Guangzhou, China). Quartz fiber (420 μM O. D.) was obtained from Scitlion Technology Co., Ltd (Beijing China); bovine serum was purchased from Sigma Aldrich (Shanghai, China) and human urine was obtained from Centre for Disease Prevention and Control of Guangdong Province (Guangzhou, China). All the plants used in this study were cultivated from seeds in the plant growth chamber (Conviron A1000, Canada).

### Synthesis of the PBA-functionalized CNTs

Firstly, the raw sample of CNTs was refluxed in nitric acid for 11 h, and the carboxylated CNTs were filtered and washed with deionized water until pH 7, and dried in vacuum oven. Then 300 mg of carboxylated CNTs were stirred in 60 mL of a 20 : 1 mixture of SOCl_2_ and DMF at 70 °C for 24 h. After the acyl chlorination, the CNTs were centrifuged and washed with anhydrous THF six times, followed by drying under vacuum. Then the acyl-chlorinated CNTs were reacted with 100 mL diamine solution at 100 °C for 48 h. After cooling to room temperature, the amino functionalized-CNTs (A-CNTs) were obtained and washed with ethanol five times to remove excess diamine. Lastly, 100 mg A-CNTs, 0.4 mmol 4-CPBA, 0.5 mmol DIC, 0.5 mmol DIEA and 0.5 mmol NHS were dissolved in a mixed solution of dichloromethane and DMF. The reaction solution was kept stirring for 24 h at room temperature. Then, diethyl ether was added to terminate the reaction followed by filtration and washing with diethyl ether, water and methanol. The product was dried under vacuum to obtain the PBA functionalized-CNTs.

### The selectivity of PBA-functionalized CNTs

For demonstration the selectivity of the PBA–CNTs toward *cis*-1,2-diol compounds, adenosine and deoxyadenosine were used as model compounds. 2 mg PBA functionalized-CNTs was added to 1 mL solution of 1 mg mL^–1^ adenosine or deoxyadenosine. The tubes were shaken on a rotator (400 rpm) for 1 h at room temperature. Then the suspension was centrifuged and the collected PBA functionalized-CNTs was rinsed with 1.5 mL of the PBS solution (pH 8.5) for 6 times each. Afterwards, the PBA functionalized-CNTs was resuspended in 1 mL of 0.2 M acetic acid solution and eluted for 1 h on a rotator with 600 rpm speed. Finally, the PBA functionalized-CNTs was recollected by centrifugation and the eluates were collected by pipetting carefully. The eluates were used for UV analysis.

### The binding capacity of the receptor

For measurement of the binding capacity, a series of adenosine solutions (1.00, 0.50, 0.10, 0.050, 0.0010, 0.00050 mg mL^–1^) was prepared. The absorbance of the six solutions was detected to draw the standard curve. 2 mg PBA functionalized-CNTs were added to 1.5 mL of 1.0 mg mL^–1^ adenosine solution in a 2 mL plastic tube. The solution was shaken on a rotator for 12 h at room temperature to ensure the binding sites were entirely complexed with adenosine. The following centrifugation and elution procedures were as above. According to the measured absorbance of the eluent and the standard curve, the concentration of the eluate was obtained, from which the binding capacity was calculated. The binding capacity of PBA functionalized-CNTs was measured to be 50.9 ± 2.3 μmol g^–1^.

### Preparation of the probe based on PBA functionalized-CNTs

Quartz fibers (QFs) were cut into 4–5 cm segments followed by sonication in water, menthol and acetone. After sonication, the QFs were then soaked in 0.1 M sodium hydroxide for 30 min to activate the surface, and the excess sodium hydroxide was then neutralized with hydrochloric acid. Finally, the OFs were dried at room temperature.

100 mg PAN was fully dissolved with 1 g anhydrous DMF in a 1.5 mL plastic tube through 1 h sonication. 40 mg PBA functionalized-CNTs was then added to the plastic tube. Another 30 min sonication was conducted to form the dispersive slurry. The pretreated QFs were dipped into the slurry and removing them slowly, a uniform coating of slurry of PAN and PBA functionalized-CNTs with 1.5 cm length was prepared on the surface of the QFs. The QFs were dried under flowing nitrogen, and finally cured for 40 min at 120 °C, which facilitated DMF to evaporate and ensure better adherence of the coating to the QFs.

### Binding capacity of the probe

To study the advantage of the nanotube stacking of the coating, another probe based on PBA functionalized-carbon dots, which possessed nanoparticle stacking (Fig. 2C and D), was used as a reference. The synthesized method of PBA functionalized-carbon dots was referenced to Shen and Xia.^48^ Briefly, 0.2 g of phenylboronic acid was dissolved in 20 mL of ultrapure water, followed by adjusting the pH to 9.0 by adding 0.1 M NaOH under stirring, then bubbling nitrogen gas for 1 h to remove dissolved O_2_. Finally, the solution was transferred to a Teflon-lined autoclave chamber and heated to 160 °C for 8 h. TEM images and fluorescence spectra of the PBA–carbon dots are provided in Fig. S7.† The preparation of the probe based on PBA functionalized-carbon dots, including material dosage and preparation process, was consistent with the method mentioned in the previous section. The characterizations of micro-morphologies were shown in Fig. 2C and D. For evaluation of the binding capacity, each kind of probe was immersed into a glucose aqueous solution (10 μM, 1.5 mL) and then shaken on a rotator. After 60 min, the probe was removed and rinsed with deionized water for 30 s followed by drying with a Kimwipe tissue. Finally, the bound glucose on the probe was then eluted in 1.5 mL 0.2 M acetic acid solution, and the concentration of glucose was detected by GC-MS.

### Comparison of extraction performance with other commonly used biological probes

PDMS and C18 commercial probes (both 45 μm in thickness) were obtained from Supelco Inc (Shanghai, China), and a further CNTs probe without PBA modification was prepared through the same method mentioned above in our lab. The concentration of glucose used was 50 μM. All the experimental parameters and relative operating process were the same as for the PBA modified CNTs probe measurements.

### Evaluation of the performance in PBS solution

For glucose assay in PBS solution, a series of glucose solutions (dissolved in PBS, 1.0, 5.0, 10.0, 20.0, 50.0, 100.0 μM) was prepared in a 2 mL brown vial with cap gasket (polytetrafluoroethylene). The procedure of introduction and fixation of the probe in PBS solution was as follows: a steel needle of a hypodermic syringe head was pierced into the cap gasket to create a hold. The end of the QFs, which is the opposite end of the coating, was then inserted into the cap gasket. Afterwards, the cap with QFs was screwed (Fig. S8†). The extraction was performed on a rotator with 400 rpm speed for 20 min. After extraction, the cap was removed and the probe was then rinsed with deionized water for 30 s and dried with a Kimwipe tissue. Subsequently, the probe was immersed into the glass vial (250 μL 0.2 M acetic acid) and eluted for 30 min (optimized in Fig. S9†) on a rotator with 600 rpm speed. The eluent was processed with a simple derivatization prior to introduction into GC-MS for glucose determination. For multi-carbohydrate assay in PBS solution, a series of carbohydrate solutions, containing ribose, rhamnose, mannose, glucose and galactose (dissolved in PBS, 0.50, 2.5, 5.0, 10.0, 20.0 μM), was prepared. The assay procedure was the same as for the glucose solutions.

### *Ex vivo* glucose assay in bovine serum and human urine

The bovine and human urine was stored at –80 °C prior to analysis. For assay in bovine serum sample, 15 μL thawed bovine serum was diluted to 1.5 mL PBS solution in a 2 mL brown vial. For assay in human urine sample, due to the trace glucose contained in urine, 1.5 mL thawed human urine was directly transferred into 2 mL brown vial by pipetting without further dilution. The probe was then directly immersed into the serum or urine sample for glucose assay without any expensive enzymes and tedious pretreatment procedure, and the assay procedure was the same as that in the previous section.

### *In vivo* carbohydrate assay in plants

The customized hollow steel needle was stabbed into the leaf or stem of the plant to a depth of about 2 cm. The probe was then inserted into the needle and reached to the end of the needle. Subsequently, the needle was carefully withdrawn back to let the probe be exposed in the plant tissues. After a certain duration, the needle was put back into the plant under the guidance of the probe to a depth of about 1.5 cm. Then, the probe was withdrawn from the needle, and the needle was removed. The total sampling duration was controlled to be 30 min. The probe were then rinsed with deionized water three times (60 s for each time) and dried with a Kimwipe tissue. Subsequently, the probe was immersed into a glass vial (250 μL 0.2 M acetic acid) and eluted for 30 min on a rotator with 600 rpm speed. The eluent was processed with derivatization and then introduced into GC-MS for carbohydrate determination.

### GC-MS analysis

The eluent for carbohydrate analysis needs a simple derivatization prior to GC-MS analysis. Briefly, 2% 0.2 mL sodium borohydride solution (dissolved in aqueous ammonia) was added into the eluent. After reaction for 20 min at 40 °C, 0.4 mL acetic acid, 0.3 mL methylimidazole and 1 mL acetic anhydride was added to the solution followed by another 10 min reaction. Finally, the carbohydrate derivatives were dissolved in 500 μL dichloromethane. The carbohydrate detection was performed on an Agilent 6890N gas chromatograph equipped with a MSD 5975 mass spectrometer (GC-MS) and electron-impact ionization (EI). A split/splitless-type injector was used for sample introduction. Chromatographic separation was carried out with a HP-5MS capillary column (30 m × 250 μm × 0.25 μm, Agilent Technology, CA, USA). The inlet temperature was 240 °C, and the oven temperature programs were as follows: the initial oven temperature was 140 °C (held for 0.5 min), ramped at 30 °C min^–1^ up to 190 °C (held for 5 min), and ramped at 2 °C min^–1^ up to 210 °C (held for 2 min). Helium was used as the carrier gas at a constant flow rate of 1.2 mL min^–1^. The MSD was operated in the electron impact ion (EI) mode with a source temperature of 230 °C. The electron energy was 70 eV and the filament current was 200 A.

### Biochemical analyzer for glucose assay

To evaluate the feasibility of the proposed probe for determination of glucose, the concentrations of glucose in human serum were assayed by an enzymatic (hexokinase) test using a PUZS-300 automatic biochemical analyzer as referenced by Shen and Xia.^48^ Briefly, the sample was firstly added into the sample cup, which was then placed in the sample frame for measurement after setting the parameters. Under the catalytic effects of hexokinase, glucose and adenosine triphosphate (ATP) can react and form glucose-6-phosphate and adenosine diphosphate (ADP). The former can dehydrogenize and form 6-phosphate glucose acid in the presence of glucose-6 phosphate dehydrogenase. At the same time, nicotinamide adenine dinucleotide phosphate (NADP) is reduced and forms nicotinamide adenine dinucleotide phosphate (NADPH). The production rate of NADPH is proportional to the concentration of glucose, which can be monitored by the absorbance at 340 nm and so allow measurement of the glucose concentration.

## Supplementary Material

Supplementary movie

Supplementary information
