# Supplementary material for: A novel probe based on phenylboronic acid functionalized carbon nanotubes for ultrasensitive carbohydrate determination in biofluids and semi-solid biotissues
Source: Chem Sci. 2015 Nov 19;7(2):1487–95. doi: 10.1039/c5sc03992d (PMC5975936; doi:10.1039/c5sc03992d)
Supplement: Supplementary file 2 [file SC-007-C5SC03992D-s002.pdf]

Electronic Supplementary Material (ESI) for Chemical Science.
This journal is © The Royal Society of Chemistry 2015

Supporting Information

A novel probe based on phenylboronic acid functionalized carbon nanotubes for ultrasensitive carbohydrate determination in biofluids and semi-soild biotissues

Guosheng Chen, Junlang Qiu, Jianqiao Xu, Xu'an Fang, Yan Liu, Shuqin Liu, Songbo Wei, Ruifen Jiang, Tiangang Luan, Feng Zeng, Fang Zhu* and Gangfeng Ouyang*

Table S1. Compared the performances of the proposed probe with the previous sensors based on phenylboronic acid for glucose assay.

Probe	Detection principle	Real sample	Linear	Respond range /mM	LOD/μM	Ref
CdSe/ZnS QDs[a]-BBV[b]	Fluorescence	no	Yes	2.5-20	/	1
CdSe/ZnS QDs[a]	Fluorescence	no	Yes	0-20	100	2
Graphene QDs[a]	Fluorescence	Rat brain	Yes	0.1-10	5.0	3
CNTs and PBA	Fluorescence	no	No	5.0-30	/	4
Copolymer gel-PBA[c]	Swelling	no	No	2.7-27	/	5
Copolymer gel-PD[d]	Swelling	no	No	2.7-27	/	6
Polymer brushes	Swelling	no	No	0.1-25	/	7
PBA[c]@PCCA[e]	Diffraction	no	No	1-100	50	8
CNTs-PBA@PAN	Couple with GC-MS	Bovine serum, human urine and plant tissues	Yes	0.001-0.1	0.12	This work

[a]QDs: quantum dots. [b]BBV: boronic acid substituted bipyridinium. [c]PBA: phenylboronic acid. [d]PD: phenylborate derivative. [e]PCCA: Polymerized crystalline colloidal arra

Supplementary Figures

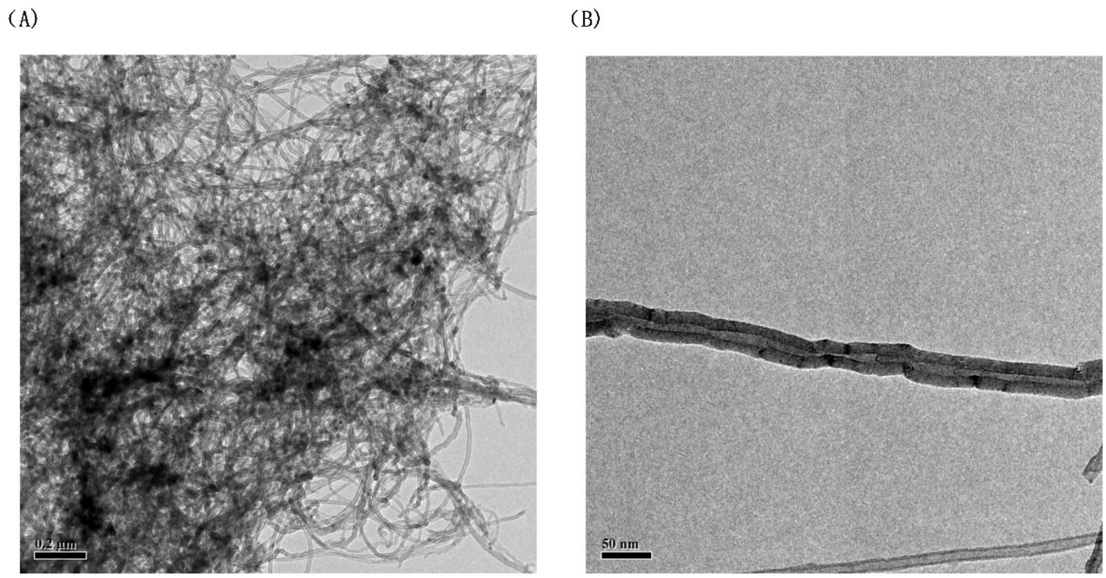

Figure S1. The transmission electron microscopic images of the PBA-functionalized CNTs at different magnification.

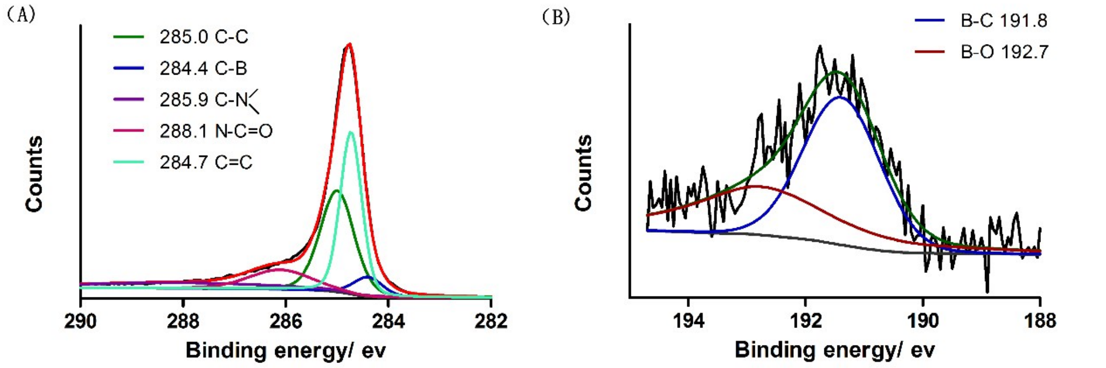

Figure S2. XPS high resolution survey scan of C 1s (A) and B 1s (B) of the PBA functionalized-CNTs.

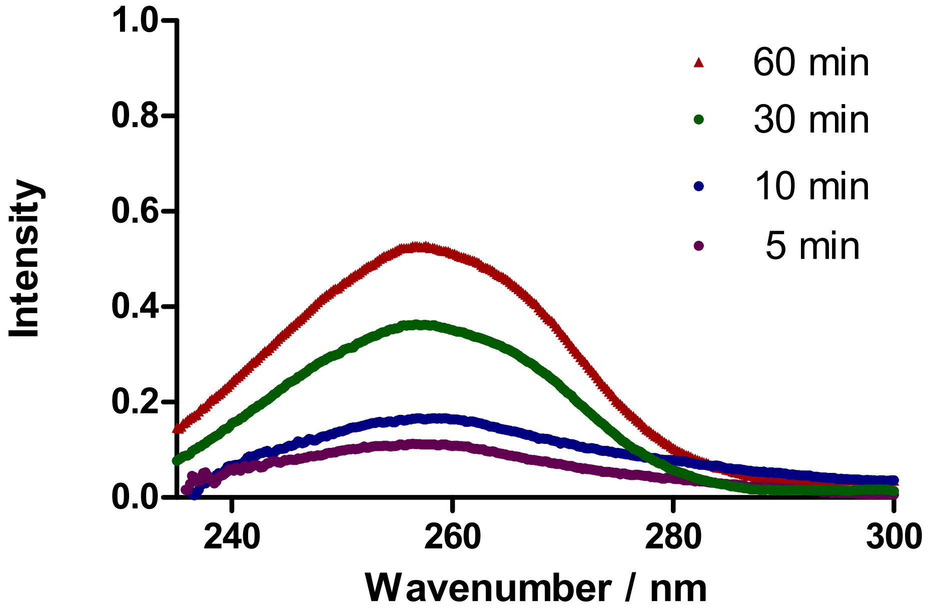

Figure S3. The UV intensities of the eluent of PBA functionalized-CNTs exposed to adenosine solution (1mg/mL) with different exposure times.

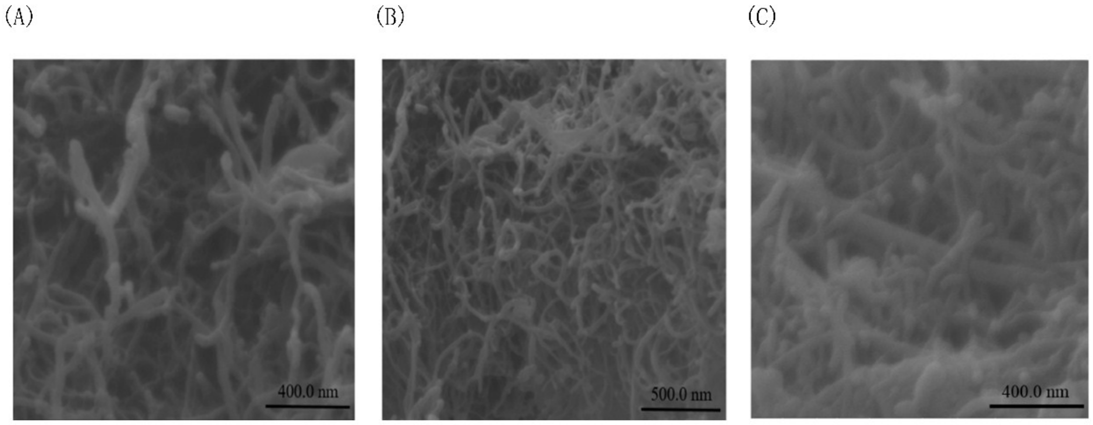

Figure S4. The 3D interconnected porous architecture in the surface (A and B) and cross section (C) of the nanotube (PBA functionalized-CNTs) based probe.

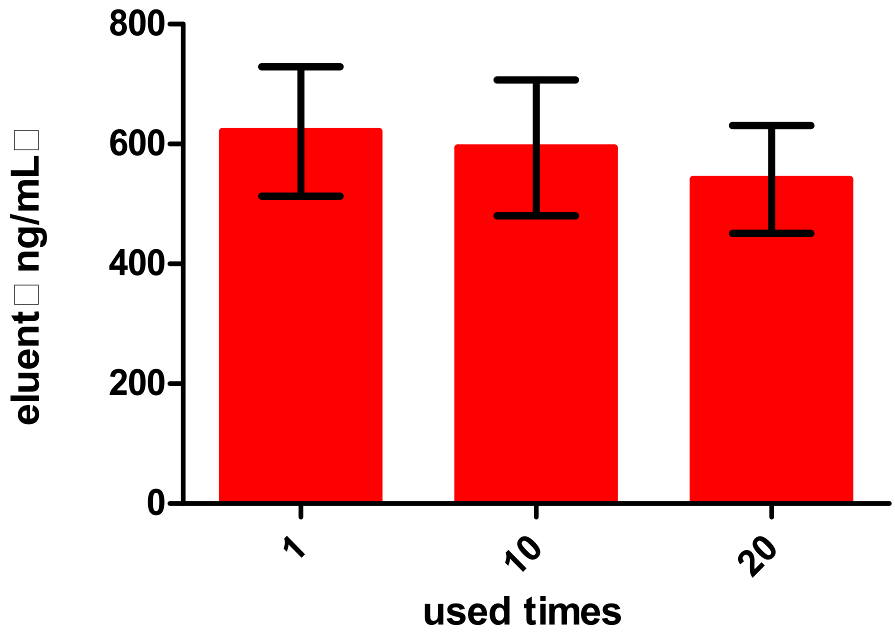

Figure S5. The performances of the probe used 1 time, 10 times and 20 times in PBS solution (50 μM glucoses)

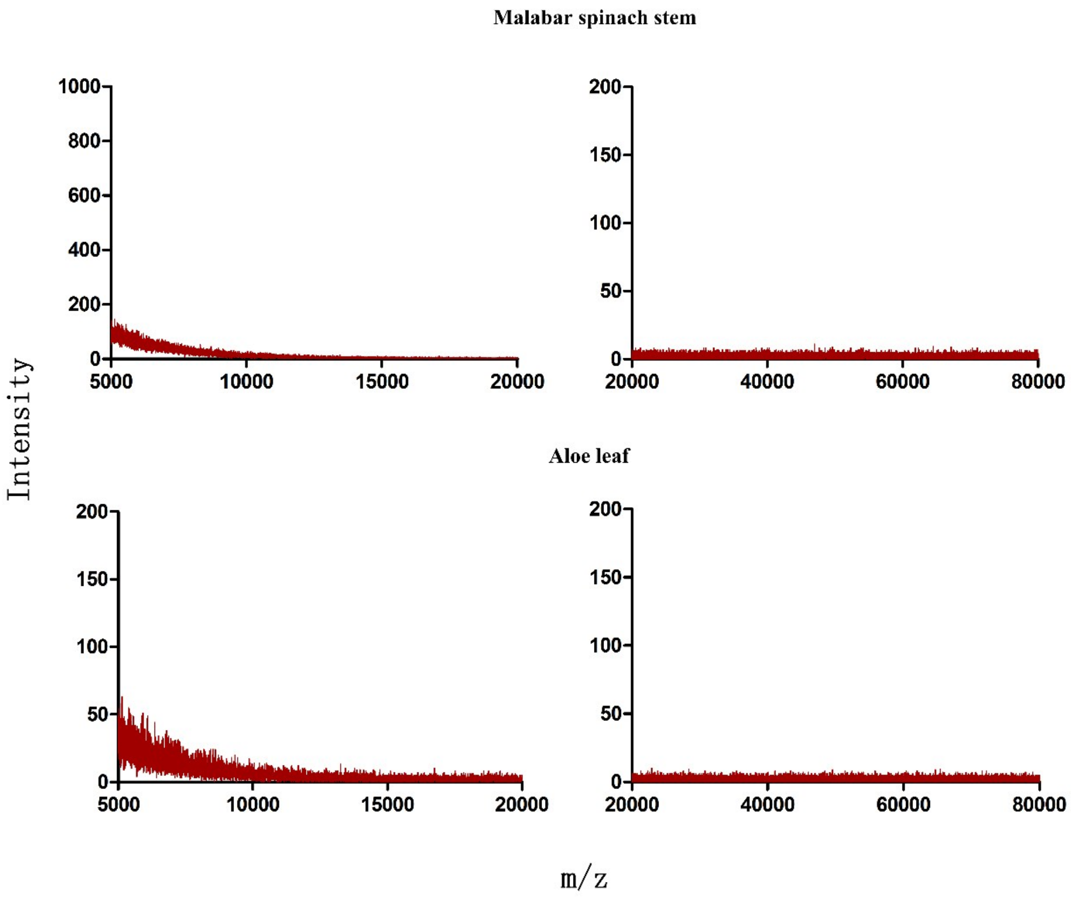

Figure S6. No macromolecules were observed through MALDI-TOF MS in the eluent of probe exposed in aloe leaf or Malabar spinach stem for 30 min.

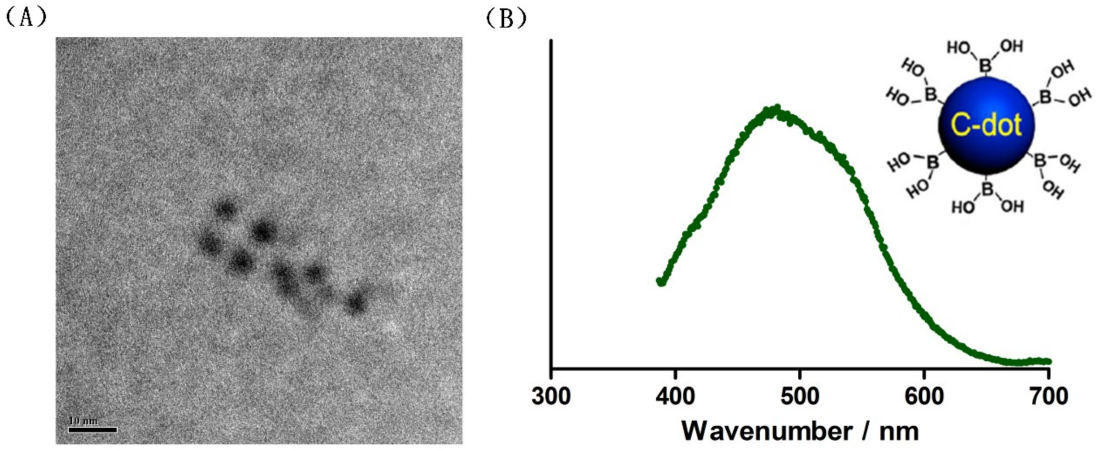

Figure S7. TEM image and fluorescence spectra of the PBA functionalized-carbon dots.

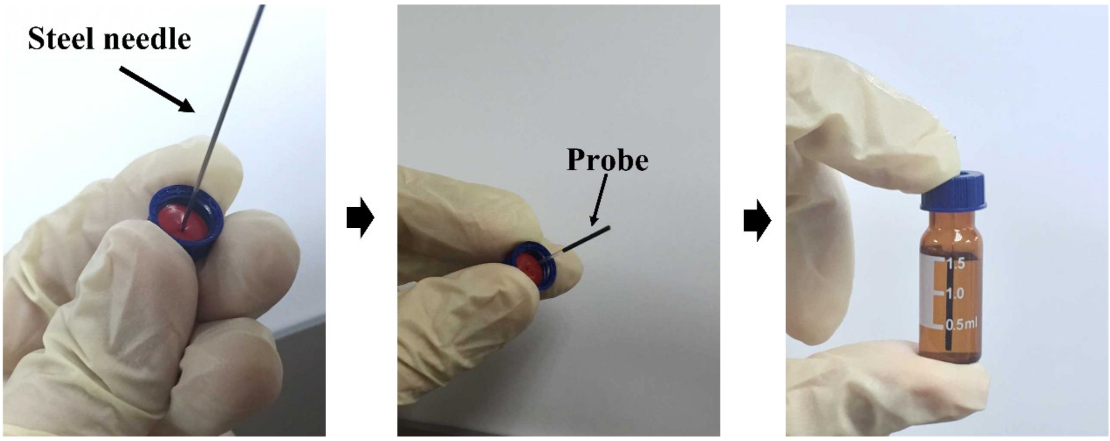

Figure S8. The procedure of introduction and fixation of the probe in PBS solution

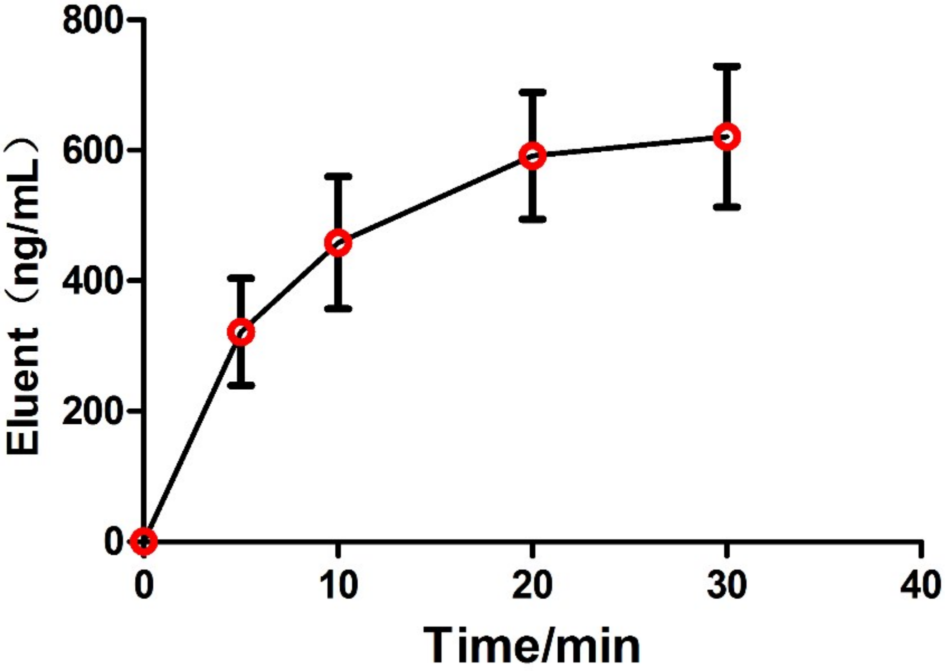

Figure S9. The concentrations of glucose in the eluents under different elution times (exposure time: 20 min, glucose in PBS solution: 50 μM)

Supplementary Reference

[1] B. David, G. Cordes, S. Bakthan, *Angew. Chem., Int. Ed*. 2006, **45**, 3829-3832.c

[2] R. Freeman, L. Bahshi, T. Finder, R. Gill, I. Willner, *Chem. Commun*. 2009, 764-766.

[3] Z. Qu, X. Zhou, L. Gu, R. Lan, D. Sun, D. Yu, G. Shi, *Chem. Commun*. 2013, **49**, 9830−9832.

[4] K. Yum, J-H Ahn, T. P. McNicholas, P. W. Barone, B. Mu, J-H. Kim, R. M. Jain, M. S. Strano. *ACS Nano.* 2012, **6**, 819-830.

[5] A. Matsumoto, K. Yamamoto, R. Yoshida, K. Kataoka, T. Aoyagi, Y. A. Miyahara, *Chem. Commun. 2*010, **46**, 2203-2205.

[6] A. Matsumoto, R. Yoshida, K. Kataoka, *Biomacromolecules* 2004, **5**, 1038-1045.

[7] N. Fortin, H.-A. Klok, *ACS Appl. Mater. Interfaces* 2015, 7, 4631-4640.

[8] S. A. Asher, V. L. Alexeev, V. Goponenko, A. C. Sharma, I. K. Lednev, C. S. Wilcox, D. N. Finegold, *J. Am. Chem. Soc*. 2003, **125**, 3322−3329.
